# Authentication of Patients and Participants in Health Information Exchange and Consent for Medical Research: A Key Step for Privacy Protection, Respect for Autonomy, and Trustworthiness

**DOI:** 10.3389/fgene.2018.00167

**Published:** 2018-06-01

**Authors:** Atsushi Kogetsu, Soichi Ogishima, Kazuto Kato

**Affiliations:** ^1^Department of Biomedical Ethics and Public Policy, Graduate School of Medicine, Osaka University, Suita, Japan; ^2^Department of Bioclinical Informatics, Tohoku Medical Megabank Organization, Tohoku University, Sendai, Japan

**Keywords:** authentication, consent, health information exchange, eHealth, rare disease, data sharing, secondary use, biometrics

## Abstract

Genome and other data are already being used in areas including cancer and rare diseases. Data-sharing and secondary uses are likely to become much broader and far more extensive; thus, obtaining proper consent for these new uses of data is an important issue. Obtaining consent through online methods may be an option to overcome the problems associated with one-off, paper-based informed consent. When the process of obtaining consent takes place remotely, authentication must be assured. Patients may also choose to store some of their own information online, such as genetic information, and allow healthcare professionals to access this data. In this health information transfer and exchange process, it is vital that anyone accessing this information be correctly authenticated to protect patients' privacy. In this article, we first clarified that authentication has two roles: i.e., not only to prevent impersonation but also to prove intent, which is a vital step to ensure that medical research and health information exchange are conducted ethically. We then set out methods of authentication. As a result, we were able to make suggestions about the requirements for authentication and a possible method of authentication for these purposes. We considered problems of biometrics and recommended two-factor authentication without biometrics as a workable solution. However, three-factor authentication including biometrics seems likely to be used once biometrics become more common.

## Introduction—new initiatives in the medical treatment and research

The era of big data is coming to medicine. Genomic analysis is being applied clinically, contributing to fields such as pathophysiology and molecular targeted drugs. One of the means to utilize genome data effectively is a biobank, which involves an unprecedented number of research participants that includes patients and the normal population depending on the project design. Thus, ethical issues such as protection of privacy have expanded. In the clinical setting, enormous amounts of electronic health information have been accumulated because of the spread of electronic medical records. As smart devices have proliferated, it has also become possible to gather health information from them. Increasing data, not only in the clinical setting, but also in the related medical researches, makes its management more difficult and it will be impossible to protect the rights of research participants unless the data management is performed reliably. Patient-oriented information provision and interactive research using electronic platforms are also under way. By analyzing big data collected from these sources using artificial intelligence, new knowledge will be created. Precision medicine, which approaches disease treatment and prevention considering individual patients' variation in genes, environment, and lifestyle, has been proposed as a framework for medical treatment and research in the future (Adams and Petersen, 2016). In this era of precision medicine, eHealth—the use of information and communication technology (ICT) for health—will become indispensable. However, there are many problems related to eHealth, including security, informed consent for data-sharing and secondary use, standardization, structuring, and deidentification (The Global Alliance for Genomics and Health, 2016; Zarate et al., 2016). Such new ethical issues are inevitable to establish a relationship of mutual trust between research participants and researchers.

This next-generation framework is either imminent or already in use, e.g., in research and treatment of some cancers and rare diseases. The number of patients with rare diseases is small, and only limited data can be collected from any one hospital or region. Therefore, it is important to connect multiple hospitals and areas, or even cooperate internationally, to gather more data. In these areas, work has already started on an international information-sharing framework (The Global Alliance for Genomics and Health, 2016). For example, Japan's Initiative on Rare and Undiagnosed Diseases (IRUD) was launched in 2015 to support research on rare diseases, and the document outlining the initiative (Adachi et al., 2017) mentions the establishment of a database that could be shared internationally. In this and other related projects, how to obtain consent is an important issue, especially for new data-sharing or secondary uses of data. Conventional paper-based consent is limited, and it may be necessary to use new methods of obtaining consent. In Japan, paper-based informed consent is still required, in line with government guidelines^1^. However, obtaining consent through online methods, including when dynamic consent is implemented online, is now attracting attention for its potential utility, such as its interactivity and continuity (Budin-Ljøsne et al., 2017). Its use will enable patients to change or remove their consent, or consent to additional healthcare services and medical research. By assuring continuity and interactivity, such a new consent system may ensure that people can participate in research more securely. However, ethical problems may be caused, such as impaired autonomy, depending on how the system will be used.

In rare disease research, several initiatives have used a new approach to providing patient-centered information. One of these is RUDY, a study in rare diseases of the bones, joints, and blood vessels organized by a research team at the University of Oxford, which uses a patient portal (Teare et al., 2017) that allows patients to enter their own information online. It also recognizes and collects subjective clinical phenotypes or health data that have not so far been examined, but will become important data. This patient-driven information provision is consistent with the current trend for patient-centered health information management. In the United States, this concept has already been widely recognized, with the spread of tools that make it easier for patients to download and share their medical records with members of their healthcare team. Examples of these initiatives include Blue Button (Hogan et al., 2014). However, this move has not yet happened on a nationwide scale in Japan and probably also elsewhere, although some local initiatives are working in this direction. In implementing any “next-generation” healthcare system, this transformation is very important^2^. It is expected that increasingly, patients themselves will also provide medical information such as medical records and even genetic information online (Kirkpatrick et al., 2015).

Thus, electronic methods such as obtaining consent through online methods and health information exchanges are expected to contribute to changes in the medical research framework. In addition, ICT can facilitate the research participants' engagement, empowerment, and mutual communication with the researchers. However, using electronic methods can lead to various ethical issues. In this article, we focus on authentication, which is one of the most important ethical issues associated with electronic methods. We clarify the roles of authentication, set out methods of authentication, and finally provide suggestions for authentication requirements and a possible method of authentication.

## Issues associated with eHealth—authentication

In dealing with the problems with eHealth as mentioned above, security is an essential condition for electronic healthcare systems. If security is not established, information leaks from the system and causes invidious damage to the privacy of research participants, including the possibility of discrimination in various ways, e.g., being denied health insurance or employment based on illness or genetic information. If the possibility of loss of privacy is clear in advance, research participants cannot participate in research with confidence. In other words, the security problem can be said to not only have a technical aspect but also an ethical aspect. Encryption can be used to protect data but relies on authentication (Heatherly, 2016). However, the importance of authentication is often not sufficiently recognized, especially in healthcare services and medical research (Li et al., 2014). For example, the authentication of most current medical research using online methods employs only passwords. The patient must be certified to use the online service. It should be noted that authentication is necessary both at the time of consent and at the time of login after consent and registration. The latter case is when the registered user returns to the system. On those occasions, if other people can access the service as that person, they will be able to see all of the information submitted previously. If they are then able to alter the consent provision, that creates another risk. This means that security is based on authentication.

Another role of authentication is to prove intent. It is essential to show the research participant's intention to join the research project when their details are entered into the electronic research system because accessing the system without sufficient intention could violate the principle of autonomy. Ethical issues need to be considered carefully for proper authentication. Proof of intent has traditionally been the role of the signature. In conventional informed consent, signing is considered to express agreement. In cyberspace, entering information based on the user's personal details (e.g., about his/her own identity) is considered to prove intent at the time of registration.

In contrast, entering a user identification and password at the time of login is, in most situations, accepted tacitly as an alternative. With the increase of the use of ICT, even simpler procedures may be preferred (e.g., the use of fingerprints for smartphones). Such simple and passive procedures, however, cannot be used to demonstrate intent. They may considerably increase the risk of agreement being given by the participants without careful consideration of the meaning and consequences of their decision, particularly concerning the extensive use of health and medical data. In other words, ethical conduct of medical research and procedures cannot be guaranteed. Therefore, it is important to use positive actions, such as entering a user identification and password as a means of authentication even at the time of login. It is not clear whether this type of authentication could satisfactorily replace a signature in informed consent to medical treatment and research. Views will differ over time and in different places, but at least for now, a simple action such as a click should not be considered sufficient.

## Authentication methods

**Figure 1:**
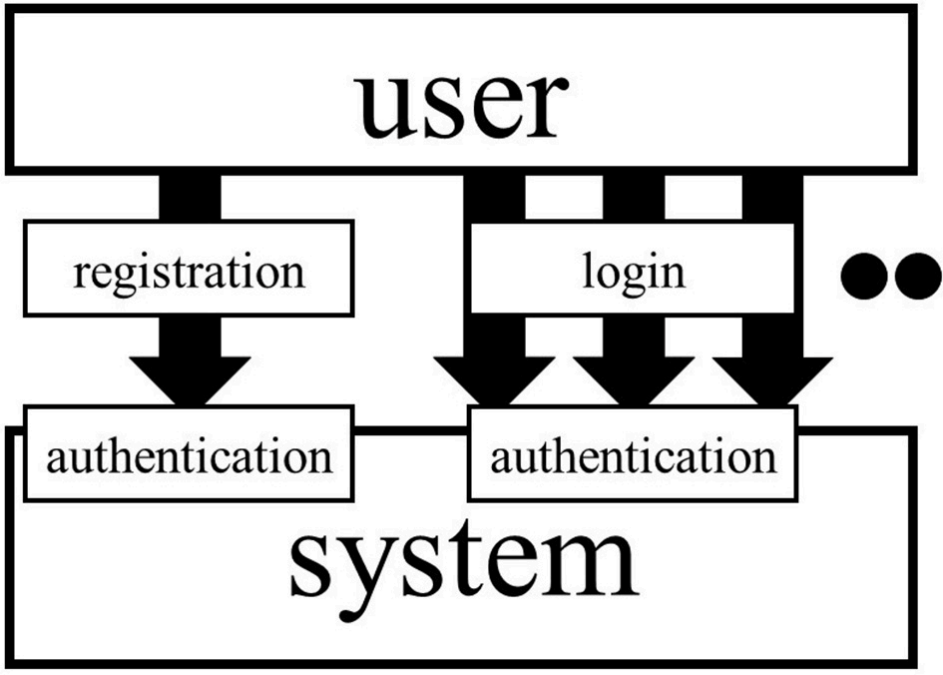
Registration and login. The user needs to be registered before using systems. They have to login when they use systems. Separate considerations are necessary for authentication at the time of registration and login.

Different methods are considered necessary for authentication at the time of registration and login (Figure 1). Login is when a registered person enters the network again. At the time of registration, it is necessary to check that the person trying to register is a research participant, e.g., that they have a rare disease. In RUDY, patients provide information about their healthcare institutions or doctors, and researchers make inquiries to check this is accurate (Teare et al., 2017). Participants can also be authenticated by uploading a medical certificate or sharing their medical records with researchers.

Login provides a more serious authentication problem because others could impersonate the user, which may result in direct information leakage. Login must include a process of authentication as a registered participant. There are broadly three ways to do this: (1) what you know, (2) what you have, and (3) what you are (Ogorman, 2003).

“What you know” authenticates users based on something only they know, such as a password or secret question. If this can be obtained or guessed by other people, they can impersonate the user. This risk increases if users choose a simple password that is easy to guess, or one that is the same as for another service. As passwords become more complicated, however, and the number of passwords used increases, it becomes more difficult to remember them (Ogorman, 2003). Even a complicated password may be obtained by keylogging.

“What you have” authenticates users based on something they own, such as a device. Users register devices in advance, ensuring that they cannot be used by anyone else. The user with that device is then authenticated. An alternative is to use a token that generates a one-off password. This type of method can be inconvenient, since users can only use preregistered devices, and loss or theft of the registered device could be a security risk (Ogorman, 2003).

Finally, new technology is allowing biometric authentication, or “what you are” systems (Jain et al., 2004). These systems authenticate users via fingerprints, faces, ears, voice print, or iris. The guidance on the use of electronic consent released by the US Food and Drug Administration included biometrics as an authentication method^3^. However, biometric information itself is sensitive and has an inherent problem that it cannot be changed if leaked. Therefore, it needs to be closely guarded (Natgunanathan et al., 2016). Biometric authentication still suffers from problems of accuracy and cost, although it has improved recently. An ideal biometric system should have complete accuracy. In the real world, no such biometric system or technology currently exists (Buciu and Gacsadi, 2016). A concern about authentication systems is that they may not work well because of the system's design at no fault to the participants. In addition, if participants use smart devices with biometric authentication systems to authenticate their identity in the research system, the accuracy of the authentication is dependent on the type of smart device used. Therefore, the research system itself cannot guarantee the security of the data. This is an ethical issue. The other option is to provide biometric authentication devices to the participants. In that case, however, the costs for devices would increase in proportion to the number of participants. Thus, biometric authentication is not recommended as a single authentication process. Another concern is that replication technologies such as three-dimensional printers and sound recording may enable “cheating” of biometric authentication in the future, and systems which can authenticate accidentally, such as a fingerprint sensor, cannot be used to prove intent.

## Trust between research participant and researcher

How to establish a relationship of trust between the research participants and researchers is the fundamental issue. The existing authentication method may not be sufficient for an updated research system. The more we rely on ICT, the more difficult it will be to establish trust. Dependence on online communication may decrease the quantity of the information exchanged, including nonverbal communication, and lead to miscommunication. In addition, we must actively face and address the related ethical issues, including privacy, data protection, and autonomy. Unless we have the necessary measures to deal with all these issues, we cannot build a relationship of mutual trust. In authentication, security must be assured to protect privacy and proof of intent must be included to maintain autonomy. Authentication is an essential step for obtaining consent through online methods or health information exchange as mentioned in the previous section. Furthermore, in establishing a relationship of trust, it is important to outline in advance the appropriate measures in case of leakage or loss of the authentication key, such as forgetting a password.

However, it is not sufficient to consider only data management in the goal of building a relationship of trust. What would happen if research participants were required to perform an excessively cumbersome operation to ensure the strict security of the data? They may become reluctant to participate in medical research. Therefore, this method can hinder mutual trust. In other words, data management must be easy to handle for research participants. Not only security in the authentication process, but also its usability is essential (Braz and Robert, 2006). A complicated authentication process would cause user frustration and increasing the number of authentication factors would exponentially increase the potential for authentication failure (Mohsin et al., 2017). This decreased usability would result in user distrust in the technology (Braz and Robert, 2006).

## Workable solutions and future challenges

Before implementing any authentication system for an eHealth system, there is a further issue to consider: what are the requirements for authentication in eHealth? Based on the discussion so far, there are four main elements. First, it must be able to prevent impersonation. Second, it should demonstrate intent. Third, it should be able to be changed, even if a user loses or forgets the authentication key. Finally, it should be user-friendly.

Some information security experts have proposed the use of three-factor authentication, which combines the three categories of authentication described previously (Jiang et al., 2016). However, this may raise some issues, especially within the requirements set out above. The first issue is usability. Three-factor authentication currently requires multiple steps, which means it is complicated to use (Mohsin et al., 2017). This complicated authentication process decreases usability and may prevent patient from participating in medical research. The second issue is caused by the use of biometric information, which is directly linked to individuals and cannot be changed if it is leaked or lost. Therefore, it is currently not practical to apply three-factor authentication to healthcare systems. An alternative is required. These two issues need to be resolved in employing three-factor authentication: to address the first issue, we need to develop a simple three-factor authentication process such as one-step three-factor authentication (Curran et al., 2017) and to address the second issue, biometrics must necessarily become more ubiquitous. If biometrics are popularly used and the biometric information for each user is saved in their smart device and not in the Cloud, the additional risk of biometric information leakage would not emerge. Even if these two issues are resolved, biometric authentication is worth employing only as an additional factor unless an ideal biometric system is developed. Using biometrics as the third authentication factor would be acceptable because the other two authentication factors guarantee a minimum security if the biometric authentication does not work well. Therefore, a biometric system will have insufficient value in many of the current electronic communication activities in medical researches or health information exchanges for some time. However, when biometrics are widespread in society, it may become a useful additional factor in strengthening authentication processes.

At present, the most practical means is two-factor authentication, as recommended in the latest guidelines from the US National Institute of Standards and Technology (Grassi et al., 2017). Sufficient security cannot be secured by single authentication, and the problems of authentication by username and password alone cannot be ignored. Combining two or more factors is more secure. Two-factor authentication combining “what you know” and “what you have” is currently the most viable. This type of method could be used for obtaining consent through online methods and the exchange of health information, such as genetic data. However, it may be necessary to strengthen the security or reduce the risk of losing keys by using graphical passwords (Biddle et al., 2012) or some other means. The acceptability of two-factor authentication is also dependent on the type of data that are stored or exchanged in the system. For example, ordinary two-factor authentication may be acceptable for subjective clinical or health data, such as blood pressure, which is measured at home or using smartphones. However, methods with higher security may be necessary if individually identifiable data or information are exchanged and stored. Thus, biometric authentication is likely to be very useful in the future, but the method requires more discussion. The combination of “what you have” and “what you are” cannot be used to demonstrate intent, since we consider “what you know” has a primary function for this purpose. Therefore, it is necessary to find an authentication system that can achieve greater security.

It is also important that an independent assessment agency consider these ethical issues. Thus, the role of ethics committees to check the above four requirements would be helpful. Additionally, the ethics committees could check whether the medical research projects or eHealth systems have an appropriate governance mechanism to review the authentication system as new technologies emerge or evolve. In summary, the ethics committees should check the following points:

1. Does the authentication system prevent impersonation? (If needed, ask the ICT experts for advice.)
2. Does the authentication system require participants to demonstrate their intent?
3. Can the user's identifying factors for the authentication system be changed, even if the user loses or forgets their authentication key?
4. Is the authentication system user-friendly? (i.e., usability)
5. Do the medical research projects or eHealth systems have an appropriate governance mechanism to review the authentication system as new technologies emerge or evolve?

It is impossible to construct a system that can completely prevent impersonation, even in the future, because of the development of new technologies. Authentication methods should be reviewed as each new technology emerges.

## Author contributions

AK and KK: discussed the importance of online mechanisms for patient participation and came up with the idea for the work; AK: conducted the literature search and analysis, and wrote the first draft; The draft was improved through discussion and editing by all the authors, who read and approved the final manuscript.

### Conflict of interest statement

The authors declare that the research was conducted in the absence of any commercial or financial relationships that could be construed as a potential conflict of interest. The reviewer MHZ and handling Editor declared their shared affiliation.

## Footnotes

^1^Ministry of Education, Culture, Sports, Science and Technology (MEXT), Ministry of Health, Labour and Welfare (MHLW), and Ministry of Economy Trade and Industry (METI), Japan. Ethical Guidelines for Human Genome/Gene Analysis Research [Internet]. [2001, fully revised in 2004 and 2013, partially revised in 2005, 2008, 2014, and 2017. The most recent edition is only available in Japanese; cited 2017 Sep 5]. Available from: http://www.lifescience.mext.go.jp/files/pdf/n1859_03r2.pdf

^2^*EU Task Force on eHealth: Redesigning Health in Europe for 2020* [Internet]. Luxembourg, Belgium: Publications office of the European Union; 2012[cited 2017 Sep 5]. Available from: http://ec.europa.eu/digital-agenda/en/news/eu-task-force-ehealth-redesigning-health-europe-2020

^3^US Department of Health and Human Services, FDA Center for Drug Evaluation. *Use of Electronic Informed Consent: Questions and Answers, Guidance for Institutional* Review Boards, *Investigators, and Sponsors* [Internet]. Silver Spring, MD: US Food and Drug Administration; 2016 [cited 2017 Jun 28]. Available from: https://www.fda.gov/downloads/drugs/guidancecomplianceregulatoryinformation/guidances/ucm436811.pdf

## Abbreviations

IRUD: Japan's Initiative on Rare and Undiagnosed Diseases
ICT: information and communication technology.
